# Collectanea Medica, Consisting of Anecdotes, Facts, Extracts, Illustrations, Queries, Suggestions, &c.

**Published:** 1816-02

**Authors:** 


### COLLECTANEA MEDICA,

CONSISTING OF

### ANECDOTES, FACTS, EXTRACTS, ILLUSTRATIONS, QUERIES, SUGGESTIONS, &c.

RELATING TO THE

***History or the Art of Medicine, and the Auxiliary Sciences.***

Quicquid agunt medici,

Nostri farrago libelli.

### *On Hernia Congenita; from* Mr. Wadd's *Cases of diseased Bladder and Testicle.*

THE discovery of the Hernia Congenita is due almost to our own times, for, highly as we may estimate the industry of the ancients, it must be admitted that the whole of this intricate complaint was entirely unknown to them. Like most other discoveries, it was progressive; and like most others in which Mr. Hunter has been engaged, it has remained as he left it.

Mr. Samuel Sharp was the first person who remarked an hernia, in which the testicle was found in contact with the gut, or, as it was then expressed, included in one sac. Baron Haller and Mr. Pott, about the same time, endeavoured to account for this, by remarking that the original seat of the testicle is in the abdomen. Both conceived that it did not reach the scrotum till after birth, in consequence of which, that the gut was very liable to come down with it. Had this been really the case, the disease must have been not only common, but, without some unknown provision, almost universal.

It was reserved for the talents of Mr. J. Hunter to unravel this mystery; and, in my opinion, there does not exist, in the whole history of anatomical research, a discovery which does greater honour to the author. It should be recollected that no common subjects of dissection could afford any information. The inquiry was confined to male children of premature birth, and it was even necessary to examine these at various periods of utero-gestation. All this, however, was accomplished by a young man, till then scarcely known to the world, and in such a manner as to gain the acknowledgment of a veteran,* whose name resounded through Europe, who was engaged in the same research, not only at the same time, but for several months before him.

This subject is so accurately detailed by Mr. Hunter, in his account of the "situation of the testis in the fœtus," that I shall only refer the reader to that paper. There is, however, one passage which equally marks his genius, his industry, and his modesty, yet

* Haller.

I do

I do not recollect that it has been noticed by any subsequent writer. Even Mr. Laurence, in his valuable treatise, in which he has given a long and ingenious chapter on Hernia congenita, has, I believe, left it unnoticed: I mean the comparatively small size of the testicle, when found in contact with the gut. Mr. Pott speaks of the testicle as wasted, which subsequent writers have considered as the effect of absorption from the pressure of the gut; but the pressure is not much greater than in common hernia; besides which, the diminutive size of the testis will be found at an age, when the pressure must have been of much shorter duration than in others, where the testicles retain the natural size.

The following are Mr. Hunter's words: "It is not easy to ascertain the cause of this failure in the descent of the testicle; but I am inclined to suspect that the fault originates in the testicles themselves; it is, however, certain that the testicle which has completed its descent, is the largest, which is the more evident in the quadruped than in the human subject; as in these we can have an opportunity of examining the parts when we please, and can determine how small, in comparison with the other, that testicle is which has exceeded the usual time of coming down; it never descends so low as the other."

The above consideration, added to the condition of the parts in hernia congenita, very much confirm Mr. Hunter's opinion, that the detention of the testicle arises from an imperfection in the gland itself.

The cases here represented, the only two of which I have taken drawings, are sufficient to illustrate the subject. And in every other case of hernia congenita which has occurred to me, the diminutive size, and flabby texture of the testis, afford the presumption of defective organization, a circumstance of little consequence to the subject, when only one testicle is in this condition.

A rupture may have the true congenital character, though it does not make its appearance till the age of puberty, or even manhood. It frequently happens that the testicle is in its progress downwards, and arrives at the groin at the age of fourteen or fifteen. At this time, even if the testicle descends by itself, a rupture is sometimes suspected, and attributed to exertion during some juvenile sport. Nothing can be more unpardonable in a surgeon, than a negligent examination of a part submitted to him. Under circumstances of hernia at all complicated, at any age, but particularly about the period above-mentioned, the scrotum should always be examined, and any preternatural situation of the testicle will instantly be detected. It should be recollected, too, that the discovery will redound much to the credit of the examiner, who will have the reputation of discovering what was before, perhaps, kept a profound secret.

Having remarked that the congenital hernia is a discovery of modern times, and having, in the present inquiry, referred in a very general manner to most of what has been written on the subject, ject, I cannot withhold from the reader the interest I felt from one part of the controversy between Mr. Pott and Dr. W. Hunter. Mr. Pott, in his otherwise elegantly written performance, uses the word *congenial* hernia. It surprised me much, that so improper a term should pass unnoticed during the heat of the controversy, and even till Mr. Laurence published his valuable and comprehensive Prize Essay in 1807, in which, with little ceremony to a name the just pride of St. Bartholomew's, he calls the term adopted by Mr. Pott "*perfectly absurd,* as applied to this or any other rupture."

The use of the term is, however, very neatly accounted for, but by no means defended, in a candid review of Mr. Laurence's work, in the 19th vol. of the Medical and Physical Journal. Since that time Mr. Laurence has published a second edition, in which he expresses the same surprise. This shews that the perusal of periodical works ought not to be considered as mere frivolous employment; they often furnish the means of information beyond the most laborious research: for, though the present question may seem unimportant, yet, as a fact, it is valuable, and may lead to others much more so.

[In our next we shall give our analysis of the above work.]

---

The late discoveries of the British and foreign chemists threaten, if not to form a new system, at least a new nomenclature, in one branch of that most interesting science.

We need only hint to most of our readers that every acid is supposed to be a compound of oxygen and some *radical* or *base.* The former term we prefer, as the latter is also applied to the alkali which neutralizes an acid. But this ingenious doctrine, like most others in which we make premature attempts at generalizing the phenomena of Nature, has been disputed by no less a philosopher than Sir Humphry Davy. To render our history as intelligible as possible, we shall first consider the properties, as far as they are discoverable, of

Muriatic acid gas;
Oxymuriatic acid, and Chlorine and Euchlorine; and
Ionine; with a few remarks on the combination of each.

Muriatic acid was, like the other acids, supposed to be a compound of oxygen and some unknown radical. Though this radical has never been discovered by itself, being, as was supposed, always mixed with a certain portion of water, or with an alkaline or metallic base, at the same time it is admitted, that the water never can be separated, or even detected, in the manner which is easily accomplished in most other gases.

Oxymuriatic acid is said to be a compound of muriatic acid with a larger proportion of oxygen: hence its name.

But on the authority of a name illustrated by a new mode of detecting the elementary parts of substances with an accuracy hitherto hitherto unknown, we are assured that oxymuriatic acid, which, from its name, might be supposed to contain a superabundance of oxygen, must be considered a simple substance, till we can decompose it, or form it by synthesis, neither of which has been hitherto accomplished.

To this hitherto undecomposed substance Sir H. D. has given the name *chlorine*, from its green appearance. When the gas was extracted in such a manner as to supersaturate it, as some would call it, with oxygen, it might be called hyperoxygenated muriatic gas, according to the common theory; but the last-named philosopher considers muriatic gas as only a compound of chlorine and hydrogen. The hyperoxygenated muriatic acid, he admits, contains oxygen, which is easily separated. In our little judgment it might, therefore, have been as well to designate this substance *oxygenated chlorine;* but, as we have such high authority for a different term, we shall, with him, use the word *euchlorine.*

Thus, muriatic acid, though we place it first in order, is considered a compound of oxygenated muriatic acid, or chlorine and hydrogen.

Chlorine then, or, as other chemists have called it, oxygenated muriatic acid, is a simple substance, and euchlorine a combination of chlorine with oxygen; and it must be admitted that all the facts brought forward by either party are explicable by either theory. This may, by some, be considered as derogatory to true philosophy; but to us it appears the triumph of improved science, when men hold different opinions, yet none accuses another of distorting facts in support of his own.

Having thus given a general view of these discoveries, and the different names by which their products are designated, we must introduce our readers to another substance and another name.

"The substance in question, *Iodine*, is prepared from kelp by a very simple process. The kelp is lixiviated with water, and the solution evaporated to dryness. This dry salt is put into a tubulated retort, with a short neck fitting into a large globular glass receiver, leaving room for the air to escape: strong sulphuric acid is poured on the salt through the tubulare of the retort, a violent effervescence ensues, and a violet coloured gas is driven off in abundance, which condenses on the receiver into shining crystallized spiculæ of a metallic appearance, somewhat resembling very fine plumbago. This substance is *iodine*, so called from the *violet* colour (ἰώδης) which it assumes when in vapour, and may be washed out of the receiver with water.

"Instead of using the entire saline contents of kelp, the solution may be boiled down and crystallized, and the mother water of these first crystals, separately evaporated to dryness, will yield the iodine more abundantly than the whole salt. Neither the first crystals of the solution of kelp, nor the residue insoluble in water, will afford any iodine.

"Iodine is an opake shining solid, permanent at a moderate temperature. When heated to about the temperature of boiling water it totally evaporates, forming a beautiful violet-coloured vapour, which immediately again condenses unchanged on the cooler part of the vessel.

"The chemical properties of the iodine hitherto discovered are very curious. It possesses in a high degree the electrical properties of oxygen and oxymuriatic acid, and when combined with hydrogen it forms a peculiar *acid* very soluble in water, capable of assuming the gasseous form, and bearing the same relation to iodine that muriatic acid does to chlorine. The action of the different reagents on iodine will be the easiest understood by the reader by bearing this relation in mind.

"When dry iodine and phosphorus are placed in contact, a reddish-brown substance is produced, and no gas is given out. When just moistened, copious acid fumes appear, which form a permanently elastic acid gas. The same acid is produced in liquid solution when the phosphorus and iodine are combined under water. In either case phosphorous acid is generated, together with the peculiar acid of iodine. If the iodine much exceeds the phosphorus, a red insoluble compound of the two is produced; but when the proportions are properly adjusted, the whole resolves into a limpid acid liquid, containing only phosphorous acid and the acid of iodine dissolved in water. These may be separated by distillation. The first liquor that condenses is mere water; but when the contents of the retort become very concentrated, the acid of iodine distils over, and at last only phosphoreous acid remains, which gives out abundance of phosphuretted hydrogen.

"This may be explained on Sir H. Davy's theory, by supposing that dry phosphorus and iodine simply combine into a compound analogous to that of phosphorus and chlorine; but when water is present it is decomposed, its oxygen acidifying the phosphorus, and its hydrogen acidifying the iodine, in the same way as hydrogen is supposed to convert chlorine into muriatic acid.

"The acid of iodine, when gaseous, is without colour, has nearly the smell of muriatic acid, smokes when exposed to the air, is rapidly absorbed by water, and gives a beautiful purple vapour with oxymuriatic gas. This acid gas, when shaken with mercury, is speedily and totally decomposed; a greenish-yellow substance, similar to the compound of iodine and mercury, is formed; and hydrogen is evolved equal in volume to half the acid gas." See Appendix to Aikin's Chemical Dictionary.

The following is from the last number of the Philosophical Transactions.

"*Some Experiments on a solid Compound of Iodine and Oxygene, and on its Chemical Agencies.* By Sir Humphry Davy, LL.D. F.R.S.

"In the two papers containing researches on iodine, which the Royal Society has done me the honour of publishing in the Trans-

 actions, actions, I have described a class of bodies consisting of iodine, oxygene, and different bases analogous to the hyperoxymuriates. In the last of these papers, I mentioned, that I had not been able to procure any binary combination of iodine and oxygene from these compounds, neither by the method proposed by M. Gay Lussac, namely, the action of sulphuric acid on the oxyiode of barium, nor by other methods of my own institution; and that in experiments on the effects of the acids on the oxyiodes, new combinations only were formed. I have lately resumed this enquiry, and by pursuing a new and entirely different plan of operation, have at last succeeded in combining oxygene and iodine. In the following pages I shall describe the circumstances which led me to ascertain the existence of this compound, and I shall detail some experiments on its analysis and its chemical agencies.

"In the course of my researches, I observed, that when a solution of the compound of iodine and chlorine was poured into alkaline solutions, or even into certain muriatic solutions, the precipitate was an oxyiode; and this fact seemed to indicate, that iodine had a stronger attraction for oxygene than chlorine; iodine, likewise, has an attraction for chlorine; it appeared, therefore, extremely probable, that euchlorine, or the gaseous combination of oxygene and chlorine, would be decomposed by heat, and two compounds formed, one of oxygene and iodine, and the other of iodine and chlorine, or that a triple compound would be produced, from which chlorine could be easily separated, and on submitting the idea to the test of experiment, I found that I had not been deceived.

"To produce the compound of oxygene and iodine, it is necessary merely to bring the euchlorine and iodine together at the ordinary temperature of the atmosphere. As soon as the euchlorine comes in contact with the iodine, there is an immediate action; its colour changes to bright orange, and a liquid is formed. When the euchlorine is in sufficient quantity, a white substance likewise appears. By the application of a gentle heat, the orange compound of chlorine and iodine may be made to rise in vapour; and the compound of oxygene and iodine remains.

"When this compound is required to be dry, the euchlorine should be passed through dry muriate of lime (calcane) before it is admitted to the iodine. The apparatus that I have employed for producing the substance is a curved bent tube, in the form of an inverted L (˥), closed at one end, the closed leg of the tube being longest, and which serves as a retort for generating the gas; a thin long-necked glass receiver for containing the iodine; and a curved tube of smaller diameter than the first, and cemented or ground into it for conveying the gas into the receiver. The muriate of lime is placed in some dry paper in the upper part of the large curved tube; and to produce the substance from 40 grains of iodine, 100 grains of the hyperoxymuriate should be used, and four times the quantity of solution of muriatic acid of specific gravity vity about 1·105; a very small spirit lamp should be employed to generate the gas; and, to prevent explosions, the heat should be applied with great care, and only to the bottom of the tube.

"The compound of oxygene and iodine, when entirely freed by heat from the compound of oxygene and chlorine, appears as a white semi-transparent solid; it has no smell, but a strong astringent sour taste. Its specific gravity is considerable, for it rapidly sinks in sulphuric acid. When heated strongly, it decomposes, undergoing fusion at the moment, and is entirely converted into gaseous matter and iodine, leaving no residuum whatever.

"It requires for its entire decomposition a heat which is rather below the boiling point of olive oil, and there seems to be little or no increase of temperature in the process.

"Its nature is proved both by analysis and synthesis, for when euchlorine acts upon iodine, the volatile substance produced has all the characters of the body produced by the immediate action of chlorine on iodine; and when the compound I am describing is decomposed in a pneumatic apparatus, the gas formed is found to be pure oxygene, and the solid sublimate produced is pure iodine.

"I endeavoured to determine the proportions of the elements in the compound, by decomposing it in glass tubes carefully weighed, and ascertaining the loss of weight of the tube, and the volume of oxygene evolved. I have used very small quantities of the substance, but as my balance is delicate, I do not think there can be any considerable error in the results. I give those which I consider as the most accurate.

"In one experiment, 3 grains of the substance afforded a quantity of oxygene equal to 517·3 grain measures of water, and lost in weight ·68. In a second experiment, 2 grains afforded 348·3 grain measures of oxygene. In a third experiment, 1 grain yielded 191 grain measures of oxygen."

These remarks will prepare our readers for the following extract from the last number of Annals of Philosophy.

"The simple supporters of combustion, at present known, amount to three, namely, *oxygen, iodine,* and *chlorine;* and, if Ampere's hypothesis respecting *fluorine*, so ably supported by Sir Humphry Davy, be correct, it will constitute a fourth. Many important facts respecting these bodies have been lately ascertained. I shall state the principal of them in this place.

"1. *Oxygen*. This substance was raised by Lavoisier to a very high rank among chemical substances. He considered it as the acidifying principle, as the only supporter of combustion, and as capable of uniting with and modifying all other simple bodies. The modern discoveries in chemistry have deprived oxygen of a good deal of its dignity. Davy has shown that it forms alkalies as well as acids, and that many acids exist which contain no oxygen. It is not, therefore, the acidifying principle. This indeed is a doctrine which was all along maintained by Berthollet, whose sagacity in many points of chemical theory deserves the highest admiration.

"Oxygen has lost likewise the property of being the only simple supporter of combustion. For chlorine possesses that property, perhaps, in a greater degree than oxygen, with this curious exception, that charcoal will not burn in it nor unite with it. Iodine is certainly a much less perfect supporter of combustion, since the only body hitherto observed to burn in it is potassium. It is amusing to observe the awkward attempts of the French chemists to preserve for oxygen the exclusive privilege of being the only simple supporter of combusion. According to them *combustion*, in the chemical sense of the word, is very different from the meaning which it bears among the vulgar. Nothing, says Thierry, is more similar to combustion than what takes place when phosphorus is introduced into chlorine gas. We have flame, and the phosphorus disappears. Nothing, on the other hand, is more unlike combustion, than the rusting of iron in a damp place. Yet the first, he informs us, is not a real combustion, while the second is. (Annales de Chimie, xciii. p. 53.) It is surprising that these gentlemen do not perceive that they are merely altering the meaning of a word, which has been known and understood ever since mankind were acquainted with fire. The burning of phosphorus in chlorine would be called *combustion* by every person of common sense who witnessed the phenomenon. Nor is there any thing in the chemical meaning of the term which is incompatible with its application to this and many other similar cases. The rusting of iron in a damp place would never be called combustion either by the vulgar or chemists, who considered the case with attention. It consists merely in the transfer of the oxygen of water to the iron. Thenard and Gay Lussac have arranged *chlorine* and *iodine* among combustible substances, merely because they have the property of combining with oxygen. If they had placed these bodies in a class by themselves, their conduct might have been excusable; but to call them combustible is absurd; because nothing similar to combustion, in any sense of the word, takes place when they unite with oxygen. The union cannot be directly accomplished, and is far from intimate. Why should the supporters of combustion not have the property of uniting with each other? It has been long known that the simple combustibles have that property. Sulphur unites to copper with such violence as to produce both light and heat in abundance; yet nobody on that account has thought proper to class sulphur among the supporters of combustion. Neither is it a sufficient reason to class chlorine and iodine along with sulpher, that all the three unite with hydrogen and form an acid.

"The only exclusive privilege which remains to oxygen is, that it alone, or its compounds, are fit for the *respiration* of animals, and necessary indeed to preserve life. The breathing of the other supporters of combustion is almost instantly fatal to animal life.

"2. *Chlorine* is now pretty generally admitted to be a simple supporter supporter of combustion. Almost the only chemist of eminence who adheres to the old opinion is Berzelius. His opposition is founded on the supposed inconsistency of Davy's theory with the chemical canons, which he has established by a vast number of uncommonly accurate analyses. But this inconsistency, I am persuaded, he will find, on a closer examination, to vanish entirely. If this were the proper place, I think I could show that the doctrine of Davy and the canons of Berzelius agree perfectly with each other.

"In Schweigger's Journal for May 1815 (vol. xiii. p. 72) there is a long paper by Professor Hildebrandt, stating several objections to Davy's theory of chlorine. I was extremely surprised on reading this paper to find that all the objections it contained had been examined and answered long ago, and that all of them were founded on mistakes. Chlorine, he says, converts nitrous gas into nitric acid, and therefore it must contain oxygen. This was the first experiment that I tried when Davy published his theory. I found that the change here stated actually took place; but on examining my chlorine, it was mixed with common air; and upon preparing pure chlorine, I found that it produced no change in nitrous gas. Davy afterwards made the same experiment, and published it; and the fact is now well known to all chemists of precision. Another objection is, that when common salt is decomposed by the galvanic battery, the chlorine appears at the positive wire. This, so far from being an objection, is a strong argument in favour of Davy's theory. Oxygen and iodine are likewise attracted by the positive pole; so should chlorine, if it be a simple supporter of combustion.

"Another objection is, that, when metals are burnt in chlorine gas, they are converted into oxides. The fact is not so, unless water be present in the vessel; they are converted into *chlorides*, a variety of which have been described by Dr. John Davy. The other objections of Hildebrandt are all of a similar nature, and do not appear to me to be worth mentioning, as they have all been refuted long ago.

"It is amusing to observe the efforts which the French chemists have made to deprive Davy of the honour of this theory. There is a long paper, by Bidault-de-Villiers, in the Annales de Chimie (vol. xciii. p. 32), to prove that Scheele did not consider chlorine as a simple substance. The proof is most extraordinary. Scheele's opinion was not adopted by chemists in general, not even by Davy himself; therefore Scheele did not maintain it. The very name, *dephlogisticated muriatic acid*, given to chlorine by Scheele, shows us what his opinion was. Phlogiston, in Scheele's opinion, as every body knows, was hydrogen gas. If, therefore, chlorine was muriatic acid deprived of hydrogen, it is obvious that he must have considered muriatic acid as a compound of chlorine and hydrogen; and accordingly this opinion was maintained by Kirwan in his Essay on Phlogiston, on the authority of Scheele. Scheele

 says, says, in his Essay on Manganese, 'muriatic acid deprived of phlogiston, which is one of its constituent parts.' *(L'acide muriatique depouillé du phlogistique qui est une de ses parties constituantes.)** I cannot conceive any thing more explicit than this.

"There can be no doubt that it was the experiments of Gay-Lussac and Thenard, published in their Recherches Physico-Chimiques, that led Davy to form the new theory. So far their merit is conspicuous. But as they did not adopt the new theory in that work, but argued against it, nothing can be more ridiculous than to claim it after it has been established by another. If Gay-Lussac always maintained it, as he informs us, but was prevented from publicly embracing it by the authority of Berthollet, we may pity his pusillanimity, but cannot, on that account, admit his claim as the first propagator of a theory which he publicly opposed. As to M. Ampere, his posthumous claim cannot be maintained, as he published nothing whatever on the subject."

"A great number of papers on *iodine* have been published during the course of the year 1815; but after the very complete treatise on that subject by Gay-Lussac, inserted in the fifth and sixth volumes of the Annals of Philosophy, these papers cannot be expected to exhibit much novelty. The following are the only new facts that I have observed in them.

"The *iodide* of gold is a white powder. Uranium is precipitated by a hydriodate of a dirty dark colour. Link, Fischer, and Steffens. (Schweigger's Journal, vol. xi. p. 134.)

"The iodide of antimony has a dark red colour, and is soluble in water. The iodide of bismuth is orange. Its solution is not precipitated by potash. The iodide of arsenic is dark purple red, and possesses acid properties. The iodide of tellurium is dark purple-red, and forms a colourless solution with potash. Ruhland, (Ibid. p. 139.)

"Dr. Wollaston has determined the figure of the crystal of iodine to be a rhomboidal octohedron, whose axes are to each other as the numbers two, three, four. (See Annals of Philosophy, vol. v. p. 237.)

"Gaultier de Claubry and Stromeyer have ascertained that starch is the most delicate re-agent for detecting the presence of iodine. The iodine must be uncombined. Starch does not detect iodine in a solution containing hydriodic acid or iodic acid. But if an acid be poured in so as to disengage the iodine, the starch shows the presence of that substance by the indigo-blue colour which it assumes. (Gilbert's Annalen, vol. xlix. p. 140; and Ann. de Chim. vol. xciii. p. 85.)

"M. Gaultier de Claubry has analyzed sea water and several fuci from the English channel. He could detect no iodine in sea

"* Memoires de Chymie de M. C. W. Scheele, i. p. 69. I quote the French translation, that the French chemists may consult the passage, though this deprives me of Kirwan's notes."

water; water; but he found it in the following sea plants: fucus saccharinus, fucus digitatus, fucus vesiculosus, fucus siliquosus, fucus filum. (See Ann. de Chim. vol. xc. p. 75, 113.)

"I have been informed that Mr. Smithson Tennant, before his death, succeeded in detecting iodine in sea water; but I know nothing respecting the method which he followed in his investigation.

"Sir Humphry Davy has discovered a solid combination of iodine and oxygen. I place it here because the discoverer does not consider it as acid unless it be combined with water, though I entertain a different opinion from this ingenious chemist. It is obtained by exposing iodine to the action of euchlorine gas. The gas is absorbed, and a solid substance formed, consisting of two compounds; the first, a combination of chlorine and iodine; the second, of oxygen and iodine. By the application of a gentle heat, the first compound is driven off, and the second remains. Sir H. Davy gives it the name of *oxiodine*; but perhaps the term *oxiodic acid* would be more proper. It is white, and semi-transparent; has no smell, but a strong astringent sour taste. It sinks rapidly in sulphuric acid. A heat rather below 600° decomposes it. According to Davy's experiments, it is composed of

| | | |
|---|---|---|
| Iodine | 81·28 | 1 atom |
| Oxygen | 18·72 | 4 |
| | 100·00 | |

"This compound is deliquescent. It is very soluble in water. The solution reddens vegetable blues, and then destroys them. It acts upon all the metals, and combines with alkalies, earths, and metallic oxides. It unites likewise with the acids, and forms with them solid compounds, which possess remarkable properties."

